# Repertoire, unified nomenclature and evolution of the Type III effector gene set in the Ralstonia solanacearum species complex

**DOI:** 10.1186/1471-2164-14-859

**Published:** 2013-12-06

**Authors:** Nemo Peeters, Sébastien Carrère, Maria Anisimova, Laure Plener, Anne-Claire Cazalé, Stephane Genin

**Affiliations:** 1 INRA, Laboratoire des Interactions Plantes-Microorganismes (LIPM), UMR441, F-31326 Castanet-Tolosan, France; 2 CNRS, Laboratoire des Interactions Plantes-Microorganismes (LIPM), UMR2594, F-31326 Castanet-Tolosan, France; 3 Department of Computer Science, ETH Zurich, Zurich, Switzerland; 4 Swiss Institute of Bioinformatics, Lausanne, Switzerland; 5 Biozentrum, Department Biologie I, Ber. Mikrobiologie, Ludwig-Maximilians Universität Muenchen, Grosshaderner Str. 2-4, 82152 Martiensried, Germany

**Keywords:** Type III effector, *Ralstonia solanacearum*, Selection, Horizontal gene transfer, Host specificity

## Abstract

**Background:**

*Ralstonia solanacearum* is a soil-borne beta-proteobacterium that causes bacterial wilt disease in many food crops and is a major problem for agriculture in intertropical regions. *R. solanacearum* is a heterogeneous species, both phenotypically and genetically, and is considered as a species complex. Pathogenicity of *R. solanacearum* relies on the Type III secretion system that injects Type III effector (T3E) proteins into plant cells. T3E collectively perturb host cell processes and modulate plant immunity to enable bacterial infection.

**Results:**

We provide the catalogue of T3E in the *R. solanacearum* species complex, as well as candidates in newly sequenced strains. 94 T3E orthologous groups were defined on phylogenetic bases and ordered using a uniform nomenclature. This curated T3E catalog is available on a public website and a bioinformatic pipeline has been designed to rapidly predict T3E genes in newly sequenced strains. Systematical analyses were performed to detect lateral T3E gene transfer events and identify T3E genes under positive selection. Our analyses also pinpoint the RipF translocon proteins as major discriminating determinants among the phylogenetic lineages.

**Conclusions:**

Establishment of T3E repertoires in strains representatives of the *R. solanacearum* biodiversity allowed determining a set of 22 T3E present in all the strains but provided no clues on host specificity determinants. The definition of a standardized nomenclature and the optimization of predictive tools will pave the way to understanding how variation of these repertoires is correlated to the diversification of this species complex and how they contribute to the different strain pathotypes.

## Background

*Ralstonia solanacearum* is a widely distributed soil-borne phytopathogen belonging to the beta subdivision of Proteobacteria [1]. It causes lethal bacterial wilt of more than 200 plant species, including economically important crops [2,3]. Among the pathogenicity determinants of this bacterium, the Type III Secretion System (T3SS) plays a crucial role because mutants unable to produce this specialized secretion machinery are unable to cause disease on plants [4]. This T3SS ensures the direct translocation of Type III effector (T3E) proteins from the bacterium to the plant cell cytosol [5,6]. These T3E are presumed to perturb host cell processes and modulate plant innate immunity to allow bacterial infection [7].

Phylogenetic analyses of *Ralstonia* strains causing wilt diseases revealed an extensive diversity [8,9] and this group of organisms is now commonly called the *R. solanacearum* species complex (RSSC hereafter) [10]. This species complex includes strains with broad and narrow host ranges with different geographic origins. Based on phylogenetic analyses and on comparative genomic hybridization, the RSSC has been classified in four phylogenetic groups called phylotypes, which reflect their origins as follows: Asia (phylotype 1), the Americas (phylotype 2), Africa (phylotype 3) or Indonesia (phylotype 4, which includes *Ralstonia syzygii* and the banana blood disease bacterium BDB) [8,11,12]. To date, 14 strains belonging to the RSSC have been completely sequenced.

Pioneering studies have established that T3E repertoires are highly variable among strains and shape the host range of bacterial pathogens [13,14]. First exhaustive inventories of RSSC T3E using different *in silico* or experimental approaches were made in phylotype 1 strains GMI1000 [5,7] and RS1000 [6,15]. GMI1000 and RS1000 have almost identical repertoires that comprise 72 and 74 T3E for which T3SS-dependent plant cell targeting have been experimentally validated in RS1000 [6,15]. A feature of these repertoires is the existence of multigenic T3E families [7]. Functional studies have been carried out on members of the Gala family, which are proteins with F-box and Leucine Rich Repeat domains collectively required for full virulence [16-18], and members of the PopP family, which includes the avirulence proteins PopP1 [19] and PopP2, the latter possessing acetyltransferase activity [20-22]. Recently a functional analysis of the AWR family demonstrated that some AWR T3E induce cell death necrotic reactions on plants and are required for full virulence [23].

The genome sequence data from strains representative of the biodiversity of the RSSC opens the way towards understanding the evolutionary processes that structured their T3E gene repertoire. This will also provide clues towards defining what makes a given strain more aggressive than others on a specific host. However such comparative genomic approaches are actually hampered by the fact that T3E inventories in multiple strains have not been accurately established: several T3E genes have been overlooked by automatic annotation programs and/or have been incorrectly predicted. Moreover, the lack of a unified nomenclature for RSSC T3E is confusing for a non-expert since many T3E genes from RSSC strains have different names in the published literature (Pop, Avr, Brg, Rip, Hpx or Lrp proteins). This doesn’t help the already difficult task of identifying orthologous and paralogous genes in strains harboring between 46 to 71 T3E genes.

This work presents an integrative and comprehensive database for the T3E of the RSSC. This database is a compendium of manually re-annotated genes across 11 sequenced strains and ordered with a novel and unifying nomenclature. This database is publicly available for browsing and retrieving data and information. Our analyses on this particular gene set at the forefront of the interaction between the bacteria and its host, provides new insight into their evolutionary history and their potential contribution to host specificity

## Results and discussion

### Ralstonia solanacearum T3E database

#### Inventory and re-annotation of T3E genes in the RSSC

**Figure 1:**
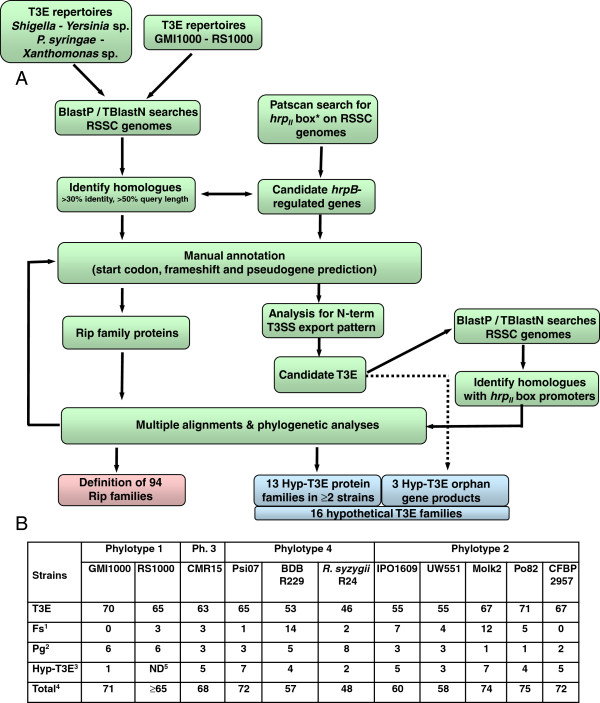
**Workflow for T3E identification in RSSC strains and main outputs of the analysis. A**. Flowchart for identification and manual annotation of T3E genes in the RSSC. **B**. T3E statistics for each of the curated strains in this study. ^1^. Number of T3E genes with a potential frameshift mutation within the coding sequence. ^2^. Number of annotated pseudogenes (incomplete or disrupted coding frames). ^3^. Number of hypothetical (candidate) T3E genes. ^4^. Total of estimated number of T3E genes (= number of T3E + frameshifted T3E + hypothetical T3E). ^5^. Not determined since the genome sequence of strain RS1000 is not available.

Our goal is to provide a comprehensive and an as exhaustive as possible inventory of T3E in the RSSC as a public database from which curated information can be retrieved. To this end, we manually curated and compiled the T3E genes from eleven sequenced strains representative of the genetic diversity of the RSSC (see Methods). The workflow of the retrieval and annotation of the T3E genes from the RSSC genomes as well as the main outputs of this analysis are shown in Figure 1. The inventory of T3E in the published RSSC genome sequences was primarily based on homology searches with the established repertoires of strains GMI1000 [7] and RS1000 [6]. Identification of additional T3E was conducted using criteria defined previously [5] to mine the GMI1000 genome: (i) homology to known T3E in other bacterial species (ii) presence of a *hrp*_II_ box in the promoter region since 52/70 T3E gene promoters harbor this *cis*-regulatory element in GMI1000 [24], (iii) existence of specific amino acid distribution biases in the 50 N-terminal domain [24]. These two latter criteria were hampered by the fact that many T3E genes have wrongly annotated start codons. Hence all the genes possessing a putative *hrp*_II_ box were inspected for potential start codon errors before being included in the T3E annotation workflow (see Figure 1). This process led to the “discovery” of twenty new T3E genes (generating 42 new gene accessions), and the re-annotation of 34% of the existing RSSC T3E genes. Altogether these changes affect 39% of the RSSC T3E dataset (841 individual entries) submitted or already present in GenBank to date.

#### Identification of T3E candidate genes in RSSC strains

A mining of the genome of nine RSSC strains from phylotypes 2, 3 and 4 for previously undescribed T3E gene families was performed based on the criteria listed above [5]. In this process, we only kept the T3E candidates strictly fitting with both criteria (ii) and (iii) described above. This search yielded 16 RSSC T3E candidates, for which T3SS-dependent translocation is not yet demonstrated. These 16 hypothetical T3E gene families are listed in the Additional file 1 as well as in the RSSC-T3E database. Most of the corresponding genes did not display homology to any other known proteins, except for families RSSC-T3E-Hyp5, Hyp6 and Hyp7 having homologues only in *Acidovorax* spp or *Xanthomonas* spp, which are both plant pathogenic bacteria.

#### *Pseudogenes*

In many cases, T3E genes appeared to have frameshift mutations or to be split into several independent open reading frames on the assembled genomes. This could be due to mutations leading to gene inactivation or, more probably, to sequence and assembly errors in the available genome sequences. It should be noted that there are important differences in terms of quality in the available assembled genomes (see Methods). In some other cases, genome sequence gaps resulted in incomplete T3E gene prediction. Many genes encoding T3E with internal repeats are often predicted as truncated or incomplete, probably due to the difficulty to assemble repeat-containing short sequence reads (Next Generation Sequencing techniques). Frameshift-mutated and incomplete T3E genes were included in the RSSC-T3E database and are distinguished by the prefix fs ('frameshift’) before the gene name. Future re-sequencing should verify the current pseudogene status of these genes.

Probable non-functional pseudogenes are also listed in the RSSC-T3E database (with the “pg” prefix, for pseudogene). These pseudogenes correspond to genes or gene fragments which are either gene remnants, open reading frames disrupted by a transposable element insertion or frameshift mutated genes confirmed after re-sequencing. The number of predicted pseudogenes varies from one to eight among the eleven strains analyzed (Figure 1B). However, the formal distinction between a pseudogene and a functional gene is difficult to establish without experimental validation [25]. In some cases, the absence of specific domains (*e.g.* RipC1_CMR15_ lacking the C-terminal half present in other RipC1 alleles) raises the question of the functionality of the corresponding protein.

#### The RSSC-T3E database interface

The dataset corresponding to the lists and expert annotation of validated and candidate T3E in the 11 sequenced strains representative of the 4 RSSC phylotypes were compiled in a web interface named “Ralstonia T3E” (https://iant.toulouse.inra.fr/T3E) designed to provide the user with a convenient and straightforward access to all the underlying data. The home page provides a synthetic table displaying the distribution of the 94 T3E gene families in the RSSC strains under the proposed nomenclature (see below). This table summarises for each strain whether a gene member is present (in single or multiple copies), absent, or is predicted as being not functional (pseudogene). A specific colour code also indicates genes with putative frameshift mutations. This information is also available as a table in the Additional file 2. The clickable T3E genes provide a link to multifasta files of the curated nucleotide and protein sequences as well as view of the corresponding DNA and protein alignments [26]. Tab-style navigation provides a link to the 16 T3E candidate genes as well as a link to different services like “ScanYourGenome” (see hereafter), Pat Scan, HMScan and Blast.

#### Proposed guidelines for the nomenclature of T3Es in RSSC strains

The recent availability of complete genome sequences for a number of RSCC strains has led to a significant increase in the rate of T3E discovery. However, the absence of a systematic nomenclature has resulted in multiple names being assigned to the same T3E gene. Some genes were named as *brg* (*hrpB*-regulated genes) [5] or *hpx* (*hrpB*-dependent expression) [27] genes based on regulation studies/screens or as *Rip* (*Ralstonia* injected protein) genes [5,6]. We propose the usage of the generic term of Rip for renaming all the T3E genes in the RSSC, a term previously used after demonstration of the translocation of these effectors into plant cells [5,6]. This new nomenclature should follow the rules defined previously for naming the *P. syringae* T3E [28]; such as: RipXY#_strain_, wherein the gene is indicated by alphabetic characters, paralogous genes in numerically characters, and the strain in subscript. The proposed attribution of this novel nomenclature to known translocated RSSC T3E is presented in Table 1 (and Additional file 3).

**Table 1:** **List of the T3E genes currently identified in the *R. solanacearum *** **species complex and proposal for a unified nomenclature**

**Proposed T3E family name**	**Representative gene member**	**Former/other name**	**Hop/Xop homolo-gues**	**Functional domain/motif or Function**	**Evidence for T3SS-dependent secretion or translocation**
RipA1	RSc2139	AWR1			RipA1 [23]
RipA2	RSp0099	RipA, Rip29, Hpx31, AWR2			RipA [5], Rip29 [6]
RipA3	RSp0846	Rip44, Hpx32, AWR3			Rip44 [6]
RipA4	RSp0847	Rip45, Hpx4, AWR4			Rip45 [6]
RipA5	RSp1024	Rip56, Hpx10, AWR5			Rip56 [6]
RipB	Rsc0245	RipB, Rip2, Hpx11	HopQ/XopQ	Nucleoside *N*-ribohydrolase	RipB [5], Rip2 [6]
RipC1	RSp1239	Rip62	XopC		Rip62 [6]
RipC2	CFBP2957 RCFBP_mp20032		XopC		
RipD	RSp0304	Rip34, Hpx25, Brg8	HopD/XopB		Rip34 [6]
RipE1	RSc3369	Rip26, Brg9	HopX/XopE		Rip26 [6]
RipE2	CFBP2957 RCFBP_mp10565		HopX/XopE		
RipF1	RSp1555	PopF1, PopF2, Rip70		T3SS translocator	RipF1 [6], PopF1 [35]
RipF2	CFBP2957 RCFBP_mp30453			T3SS translocator	
RipG1	RSp0914	Gala1, Rip53		F-box Leucine-Rich Repeats	Rip53 [6]
RipG2	RSp0672	Gala2, Rip37, Hpx20		F-box LRR protein	Rip37 [6]
RipG3	RSp0023	Gala3, Rip28		F-box LRR protein	Rip28 [6]
RipG4	RSc1800	Gala4, Rip17, Hpx15		F-box LRR protein	Rip17 [6]
RipG5	RSc1801	Gala5, Rip18, Hpx16		F-box LRR protein	Rip18 [6]
RipG6	RSc1356	RipG, Gala6, Rip13, Hpx13		F-box LRR protein	RipG [5], Rip13 [6]
RipG7	RSc1357	Gala7, Rip14, Hpx14		F-box LRR protein	Gala7 [16], Rip14 [6]
RipG8	CMR15 CMR15v4_10224	Gala8			
RipH1	RSc1386	HLK1, Rip15, Brg19	XopP		Rip15 [6]
RipH2	RSp0215	HLK2, Rip32	XopP		Rip32 [6]
RipH3	RSp0160	HLK3, Rip30, Brg18	XopP		Rip30 [6]
RipH4	Psi07 RPSI07_mp0161	HLK4	XopP		
RipI	RSc0041	Rip1			Rip1 [6]
RipJ	RSc2132	Rip22	HopZ/XopJ	Putative acetyltransferase	Rip22 [6]
RipK	CFBP2957 RCFBP_mp10024			YopJ acetyltransferase domain	
RipL	RSp0193	Rip31, Brg22		Pentatricopeptide Repeats	Rip31 [6]
RipM	RSc1475	Rip16, Brg42			Rip16 [6]
RipN	RSp1130	Rip58, Hpx26, Brg44		Nudix hydrolase domain	Rip58 [6]
RipO1	RSp0323	Rip35, Brg12	HopG		Rip35 [6]
RipO2	*R. syzygii* RALSY_mp30159		HopG		
RipP1	RSc0826	PopP1, Rip7	HopZ/XopJ	Putative acetyltransferase	Rip7 [6], PopP1 [36]
RipP2	RSc0868	PopP2, Rip8	HopZ/XopJ	Acetyltransferase	PopP2 [5], Rip8 [6]
RipP3	UW163 [GenBank accession : CAF32358.1]	PopP3	HopZ/XopJ	Putative acetyltransferase	
RipQ	RSp1277	Rip63, Hpx23	HopAA		Rip63 [6]
RipR	RSp1281	Rip64, Hpx24, Brg15, PopS	HopR		Rip64 [6]
RipS1	RSc3401	SKWP1, Rip27, Hpx37	XopAD	Heat/Armadillo repeat domain	Rip27 [6]
RipS2	RSp1374	SKWP2, Rip65, Hpx36		Heat/Armadillo repeat domain	Rip65 [6]
RipS3	RSp0930	SKWP3, Rip54		Heat/Armadillo repeat domain	Rip54 [6]
RipS4	RSc1839	SKWP4, Rip20, Hpx30		Heat/Armadillo repeat domain	Rip20 [6]
RipS5	RSp0296	SKWP5, Rip33, Hpx34		Heat/Armadillo repeat domain	Rip33 [6]
RipS6	RSc2130	SKWP6		Heat/Armadillo repeat domain	
RipS7	Molk2 RSMK02658	SKWP7		Heat/Armadillo repeat domain	
RipS8	Psi07 RSPsi07_1850	SKWP8		Heat/Armadillo repeat domain	
RipT	RSc3212	RipT, Rip25	HopC	Putative cysteine protease	RipT [5], Rip25 [6]
RipU	RSp1212	Rip59			Rip59 [6]
RipV1	RSc1349	Rip12, Hpx29, Brg17		Ubiquitin ligase domain	Rip12 [6]
RipV2	Psi07 RSPsi07_1895			Ubiquitin ligase domain	
RipW	RSc2775	PopW, Rip24		Harpin, Pectate lyase	Rip24 [6], PopW [34]
RipX	RSp0877	PopA, Rip49		Harpin	Rip49 [6], PopA [74]
RipY	RSc0257	Rip3, Brg23		Ankyrin Repeats	Rip3 [6]
RipZ	RSp1031	Rip57, Brg38			Rip57 [6]
RipAA	RSc0608	AvrA, Rip5, Brg46			AvrA [31], Rip5 [6]
RipAB	RSp0876	PopB, Rip48			Rip48 [6], PopB [33]
RipAC	RSp0875	PopC, Rip47	XopAE	Leucine-Rich Repeats	Rip47 [6], PopC [33]
RipAD	RSp1601	Rip72			Rip72 [6]
RipAE	RSc0321	Rip4	HopZ/XopJ	Putative acetyltransferase	Rip4 [6]
RipAF1	RSp0822	Rip40	HopF	PutativeADP-ribosyltransferase	Rip40 [6]
RipAF2	*R. syzygii* RALSY_20037		HopF	PutativeADP-ribosyltransferase	
RipAG	RSc0824	Rip6			Rip6 [6]
RipAH	RSc0895	Rip11			Rip11 [6]
RipAI	RSp0838	Rip41			Rip41 [6]
RipAJ	RSc2101	Rip21, Hpx18			Rip21 [6]
RipAK	RSc2359	Rip23, Hpx28, Brg36			Rip23 [6]
RipAL	UW551 RRSL_02221	Rip38		Lipase domain	Rip38 [6]
RipAM	RSc3272	Brg40			This work Additional file 3
RipAN	RSp0845	Rip43, Hpx33, Brg33			Rip43 [6]
RipAO	RSp0879	Rip50, Hpx2, Brg34			Rip50 [6]
RipAP	UW551 RRSL_04655	Rip60		Ankyrin Repeats	Rip60 [6]
RipAQ	RSp0885	Rip51, Brg35			Rip51 [6]
RipAR	RSp1236	Rip61		Ubiquitin ligase domain	Rip61 [6]
RipAS	RSp1384	Rip66, Hpx9, Brg43			Rip66 [6]
RipAT	RSp1388	Rip67, Brg48			Rip67 [6]
RipAU	RSp1460	Rip68, Hpx8, Brg45			Rip68 [6]
RipAV	RSp0732	Rip39, Hpx27, Brg39	HopAV		Rip39 [6]
RipAW	RSp1475	Rip69		Ubiquitin ligase domain	Rip69 [6]
RipAX1	RSc3290	Brg13	HopH/XopG		
RipAX2	RSp0572	Rip36, Brg14	HopH/XopG		Rip36 [6]
RipAY	RSp1022	Rip55, Hpx21, Brg37			Rip55 [6]
RipAZ1	RSp1582	Rip71			Rip71 [6]
RipAZ2	*R. syzygii* RALSY_20407				
RipBA	RSc0227, RSp0228 [pseudogene]		AvrRpm1		
RipBB	Psi07 RPSI07_mp0573			Ankyrin repeats	
RipBC	CFBP2957 RCFBP_mp30170			YopJ acetyltransferase domain & Ankyrin Repeats	
RipBD	*R. syzygii* RALSY_20184		HopAF		
RipBE	RS1000 Rip10	Rip10	XopAR		Rip10 [6]
RipBF	Psi07 RPSI07_2863		HopV		
RipBG	Molk2 RSMK00763		HopAB	Ubiquitin ligase domain	
RipBH	Psi07 RPSI07_mp1715			*Shigella flexneri* OspD family	
RipBI	CFBP2957 RCFBP_mp30113		XopX		
RipTAL1	RSc1815	Rip19, Hpx17, Brg11	TAL	Putative transcription factor	Rip19 [6]
RipTPS	RSp0731			Trehalose-phosphate synthase	Manuscript in preparation

A representative gene member for each family is provided (gene nomenclature from strain GMI1000 unless otherwise stated) with other names published in the literature. Homologues T3E from *Pseudomonas syringae* sp. (Hop) or *Xanthomonas* sp. (Xop) are indicated. The last column lists T3E for which Type 3 secretion system-dependent secretion or translocation was experimentally demonstrated.

After identifying groups of homologous genes by reciprocal best hit in the curated list of RSSC likely T3E genes, we concentrated our effort in grouping the different genes in orthologous groups and naming then accordingly. Three situation can occur: (i) a single hit (or no hit) in each strain, with conservation of synteny on the genome; (ii) a single hit (or no hit) in each strain, but with a breach of synteny for at least one of the homologous genes; (iii) multiple hits (two or more for at least one strain) in different strains.

In the first case a single orthologous group is defined irrespective of the pairwise identity between the orthologous genes. This can be exemplified by RipB a single gene present in all strains with pairwise amino acid identity ranging from 72 to 100%. Another case is RipU also a single gene present in all strains with a strict conservation of synteny, but with surprising divergent members (pairwise amino acid identity ranging from 23 to 100%). Even though it is likely that RipU has evolved different functions in the different strains, based on the likely common ancestral origin suggested by the conservation of synteny [29,30], we advocate for keeping a single orthology group.

In the second situation, an apparent single orthologous group exists but differences in synteny support a scenario of gene duplication followed by gene loss or lateral gene transfer between strains. Here we favour synteny as a ruler for ortholog definition [29,30]. This is exemplified by RipO1 and RipO2, the latter being present only in the strain R24, devoid of RipO1.

Finally when there are strains with two or more paralogous genes, again we favour the synteny rule to identify groups of orthology [29]. A careful phylogenetic reconstruction for these homologous genes across the whole species complex (Additional file 4) illustrates the accuracy of the orthology attributions [30]. These phylogenetic trees also highlighted the existence of two paralogs in several strains that clearly belong to a clade defined as an orthologous group (see Additional file 4, for RipA5, RipE1, RipF1, RipG1 and RipH2). We believe that these paralogs result from strain specific (or group of related strains) recent gene duplication. We thus choose to name these genes in a way that indicates their recent evolution: *e.g.* RipA5_1_Molk2_ and RipA5_2_MolK2_; RipF1_1_CMR15_ and RipF1_2_CMR15_ etc.…The rule of synteny is conserved since we verified that all these genes have indeed a conserved synteny (*e.g.* RipA5_1_MolK2_, RipA5_1_IPO1609_, RipA5_1_UW551_ and RipA5_1_P082_ have a conserved genomic location, as do RipA5_2_MolK2_, RipA5_2_IPO1609_, RipA5_2_UW551_ and RipA5_2_P082_).

#### Suggested name reassignment of previously characterized R. solanacearum T3E

Whenever possible the proposed new nomenclature conserves the original letter designations used in previous annotation *e.g.* RipP1 is PopP1 [19]; RipP2 is PopP2 [20]; RipAA is AvrA [31]. In the case of paralogous genes, the names are, for instance: RipG1, RipG2, …to RipG8 for the GALA gene family [16,17]; RipA1, RipA2, …to RipA5 for the AWR family [23]. In a few cases, there is evidence for recent T3E gene duplications resulting in two or more gene copies in a single given strain, *e.g.* strain Psi07 harbors 3 copies of RipG1 [17] and 2 copies of RipH2: these were renamed RipG1_1, RipG1_2, RipG1_3 and RipH2_1, RipH2_2, respectively, to differentiate them from the other RipH and RipG genes in this strain (Table 1).

In addition, a Rip name is proposed for the 9 T3E previously identified as Pop [20,32-36] or Avr [37]. The Pop designation is historical and was formerly coined when *R. solanacearum* was known as *Pseudomonas solanacearum*[38], the “Avr“ term was solely used for the AvrA avirulence protein identified in 1990 [37]. These designations can be confusing because the Pop term has also been used to name some *Pseudomonas aeruginosa* T3E [39] and AvrA also refer to an unrelated T3E from *Salmonella* species [40].

#### “ScanYourGenome” a bioinformatic tool for detecting T3E orthologs

In order to swiftly analyse the T3E content of newly produced genome sequences, we developed a protocol for the identification of putative effector candidates. This pipeline is based on a *de novo* effectome prediction using T3E models. Then each candidate is tested using different methods with decreasing stringency to assign them to the most probable known effector gene (see Methods section). This protocol was first tested on reference genomes used above for manual annotation of the T3E genes in order to calibrate the detection parameters (see Methods) before using it for predicting T3E in the recently published draft genomes of strains K60 [41], FQY_4 [42] and Y45 [43]. This analysis yielded a prediction of 60, 75 and 73 potential T3E encoding genes encoded respectively by the K60, FQY_4 and Y45 genomes, (Additional file 2). The gene model prediction takes into account possible frameshifts, also when the gene is shorter than 80% of the average length of the other alleles of this Rip gene, the predicted gene is tagged as potential pseudogene. Both frameshift and pseudogene annotations appear in the prediction. This orthology search engine and the consequent Rip assignment are available to the community for queries of draft or complete genome sequences. For shorter gene sequences a more straightforward blast is advised. The advantage of a sliding scale of orthology detection is the possibility to unequivocally assign each potential T3E gene to a specific orthologous group. Whenever a new candidate T3E gene, experimentally validated as being secreted or translocated into plant cells, will not retrieve an already labelled orthologous Rip family, this gene will be assigned the next available Rip code.

### Evolutionary dynamics of rip genes

#### Classification of paralogous rip genes

A specific feature of *R. solanacearum* T3Es is the abundance of paralogous *rip* genes in all the strains sequenced to date. Some of these paralogous genes are well represented in strains from the four phylotypes, hence they probably originated from ancient duplications in the common ancestor of these diverse strains. This was well documented for the RipG1-G8 [17] and the RipA1-A5 [23] paralogous gene families and is probably also true for RipH1-H3 and RipS1-S8. Although all strains contain members of these paralogous family, the likely ancient duplications doesn’t exclude some phylotype specificities explained by loss or more simply by recent duplications *e.g.* RipA1 and RipS6 seem to be specific to phylotype 1, RipG8 is only found in CMR15, the sole representative of phylotype 3; and RipH4 seems to be specific of the phylotype 4 strains (see Additional file 2).

A second group of paralogous *rip* genes is characterised by a smaller number (2–3) of paralogous sequences in a given strain. Phylogenetic analyses were used to estimate the evolutionary relationships between paralogues using sequence data from the 11 RSSC representative strains. We defined eight additional *rip* genes (RipC2, RipE2, RipF2, RipO2, RipV2, RipAF2, RipAX2 and RipAZ2) (Table 1 and Additional file 4). Several of these paralogous genes, such as *ripC2* or *ripO2*, seem to differ significantly from *RipC1* and *RipO2* respectively and could have originated through lateral gene transfer (see below) since homologous genes exist in other bacterial species. For the gene families present in most of the RSSC strains (*ripE2* and *ripV2*), the genes are located in each strain in a similar genomic context, an observation which also supports a common evolutionary origin. But distribution of some paralogs can be variable among strains: *.i.e.* RipE1 seems to be ubiquitously present whereas RipE2 is absent in phylotype 1 strains.

Protein sequence analyses indicated that RipAR, RipAW, RipV1, RipV2 and RipBG contain putative ubiquitin-ligase domains (see below), likewise, RipJ, RipK, RipAE, RipBC, RipP1 and RipP2 could all potentially display acetyltransferase activity (see phylogenetic tree in Additional file 5). Notwithstanding this apparent functional conservation, the sequences of these T3E genes have diverged significantly and can’t be assigned in orthologous goups. It has to be noted that the numerical identification of the two RipP1 and RipP2, and the pseudogene RipP3_GMI1000_ is used in reference to their previous names PopP1 [7,36] (RipP1), PopP2 [20,22,44] (RipP2) and PopP3 [19]. This is an exception to the previous rule as we don’t consider these to be paralogs.

#### Horizontally acquired rip genes

The detection of horizontal gene transfer (HGT hereafter) events in a given bacterial genome can be performed retrospectively through bioinformatics-based comparative analyses [45]. A frequent hallmark of genes with an extrinsic origin is the difference in GC content of these genes compared of the mean content of the host genome [46,47]. Thirteen Rip genes exhibit a mean GC% below 60% (whereas the genomic mean content in RSSC strains is 67%) (Additional file 6). In several cases, the T3E gene is physically associated with insertion sequence elements (RipAA, RipAX1, RipO2, RipE2), integrases (RipAF2) or are part of prophage sequences integrated in the genome (RipP1, RipP2, RipT, RipAG, RipAX2, RipE2, RipBD). From these observations, we can assume that bacteriophage-mediated transfer appears to be an efficient mean for lateral transfer of these T3E in the RSSC.

Phylogenetic analyses also provided interesting insights into possible HGT with other bacterial plant pathogens. For example, RipC2_CFBP2957,_ outgroup of the RipC1 gene family, could derive from the XopC T3E from *Xanthomonas* spp . Furthermore, the low GC content of *ripC2*_*CFBP2957*_ (61%) supports the hypothesis of an HGT, with the possibility of a shared common ancestor between *ripC2*_*CFBP2957*_ and *xopC*. Similar observations can be made with RipO2_*R.syzygii R24*_ (and *P. syringae* pv. *phaseolicola* HopG1), RipAF2_*R.syzygii R24*_ (and *P. syringae* HopF1), RipE1 (and *P. syringae* HopX1 and *Xanthomonas* spp. XopE), RipP1 (and *Xanthomonas* spp. XopJ), RipAX2 (and *Xanthomonas garderni* XopG and *P. syringae* HopH1) and RipH2 (and *Xanthomonas* sp. XopP), see Additional file 4. Together with RipTAL , already suspected of inter-species transfer [48,49], this analysis thus provided a total of seven T3E genes that could have been acquired through HGT.

#### Evidence of phylogenetic incongruences

**Figure 2:**
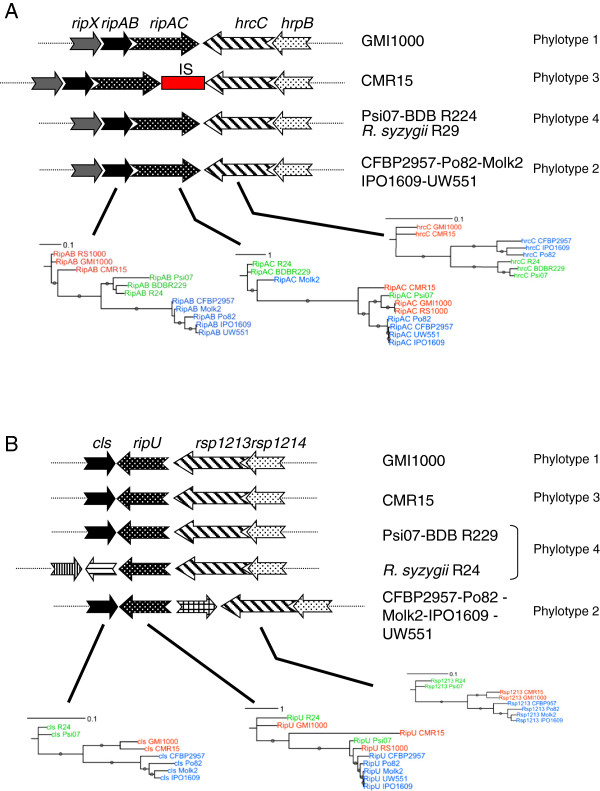
**The RipAC and RipU T3E loci are incongruence hotspots. A**. Genomic map of the *ripAC* locus in representative strains of the four phylotypes from the RSSC and phylogenetic relationships of *ripAC* and its flanking genes. Arrows of same colour symbolize orthologous genes. **B**. Similar analysis as above for *ripU*. RSSC strains are color-coded according to their phylotype goup: Red for phylotype 1 and 3; Blue for phylotype 2 and green for phylotype 4 and related strains.

Examination of the intra-family phylogenetic relationships of T3E genes distributed in the nine RSSC sequenced strains revealed in some cases incongruences with the species phylogenetic tree. This can be illustrated by individual Rip contradicting the species phylogeny like RipG7_CMR15_[17], RipD _CMR15_, RipH2_1_Po82_ and RipAX1_Po82_, which could be indicative of rapidly evolving or horizontally acquired genes (Additional file 4). Some other conflicting phylogenies can’t be directly associated with a single divergent gene. This is the case for RipI, RipU and RipAC which are present in most of the RSSC sequenced strains (especially RipAC and RipU) but with great sequences divergence (identity at the protein level falling under 30% between some RipU and RipAC alleles). The only strong evidence for them being orthologs is the fact that RipAC and RipU genes are located in two highly syntenic regions with their respective flanking genes strictly following the species phylogeny (Figure 2). This suggests that RipU and RipAC evolved faster in some strains (*e.g.* RipU_CMR15_) resulting in this particular high sequence polymorphism [50].

Another example of discrepancy between species and gene phylogeny is for RipAA. Here the increased polymorphism is correlated with the presence of a hypervariable domain consisting of Variable Number of Tandem Repeats [31].

#### Several rip genes underwent selection and recombination

After excluding from the datasets the likely pseudogenes, all Rip genes with more than 3 orthologs (75 out of 93 Rip genes) were analysed for traces of recurrent diversifying positive selection. The analysis performed here was carried out like described previously [17], except that gene phylogenies were inferred using one-ratio codon model M0 [51]. The full results are displayed in the Additional file 7. Considering that some of the datasets were rather small we concentrated on identifying Rip genes with strong indications of positive selection. This was the case for the nine following Rip genes: RipAA, RipAJ, RipAT, RipAW, RipBD, RipD, RipG7, RipH3 and RipS7 with three out of three likelihood ration tests (LRTs) for positive selection being significant (Table 2). Six out of these 9 Rip genes have an estimated proportion of sites under positive selection higher than 5%, with the highest level reached for RipAJ and RipG7, in agreement with a previous analysis [17].

**Table 2:** Rip coding sequences under strong diversifying positive selection on the protein level

***T3E gene***	**Number of strains**	**Alignment length (nt)**	**Population recombination rate,** ***N***_***e***_ ***r*** **(*P***_**LPT**_**)**^**a**^	**LRT statistic values for codon model pairs**^**b**^				**Proportions of sites in different selection regimes**^**c**^			
				**M0 vs M3**	**M1a vs M2a**	**M7 vs M8**	**M8a vs M8**	**Strict negative (ω< 0.15)**	**Relaxed negative (0.15 <ω<0.9)**	**Neutral (0.9<ω<1)**	**Positive (ω>1)**
*RipAA*	10	906	10 (0.33)	134.9	6.2	14.4	13.1	38% (ω=0.04)	56% (ω=0.48)	0%	6% (ω=2.9)
*RipAJ*	11	936	2 (0.54)	150.5	7.2	11.9	13.3	46% (ω=0.04)	46% (ω=0.49)	9%	7% (ω=3.1)
*RipAT*	9	1764	0 (0.04)	191.1	11.5	17.3	13.3	38% (ω=0.04)	48% (ω=0.54)	10%	5% (ω=3.3)
*RipAW*	6	1359	**6 (0.00)**	177.6	9.4	15.0	20.6	38% (ω=0.02)	48% (ω=0.51)	10%	5% (ω=4.9)
*RipAP*	7	2400	0 (0.22)	148.6	18.2	21.7	27.6	59% (ω=0.02)	29% (ω=0.48)	10%	2% (ω=10.0)
*RipD*	11	1971	3 (0.18)	266.5	7.2	16.7	12.7	38% (ω=0.04)	47% (ω=0.47)	9%	6% (ω=2.8)
*RipG7*	10	2016	**10 (0.00)**	561.6	28.1	37.6	43.0	46% (ω=0.04)	28% (ω=0.59)	19%	7% (ω=3.4)
*RipH3*	9	2229	4 (0.81)	145.7	19.5	29.5	25.1	29% (ω=0.07)	57% (ω=0.56)	10%	4% (ω=3.8)
*RipS7*	7	9570	0 (0.00)	329.0	30.4	34.3	42.2	48% (ω=0.03)	38% (ω=0.44)	10%	5% (ω=4.0)

^a^Values supporting evidence for recombination are shown in bold.

^b^For the presented 9 genes all three LRTs for positive selection were significant, as well as the LRT comparing M0 vs M3 supporting strong variability of selection pressure among sites. Codon models are as described in [74].

^c^Estimates of selection regimes are according to model M8 if LRT comparing M8a and M8 was significant. Otherwise, selection regimes are reported according to model M8a. For strict and relaxed negative selection, the average omega value over respective selection classes is shown. Note that percentages for the four categories do not always add up to 100% due to rounding.

Importantly, the presence of a high degree of recombination can hamper LRTs for positive diversifying selection, leading to false positives [52]. However inference of recombination can also be affected by selection forces [53,54]. This is why we systematically analysed all data for evidence of recombination (see Additional file 7 for full results). Table 2 also displays the results of tests for recombination for the nine previously identified Rip genes. Among these, only two (RipAW and RipG7) could also be affected by recombination, while for RipAA the evidence of recombination is not clear-cut. The interplay between selection and recombination was already disentangled previously for RipG7 [17], with the conclusion that there is indeed a strong likelihood of positive selection acting on this gene. Here we won’t address the question further for RipAA and RipAW but a future analysis with more allelic variants should be informative.

It is interesting to note that in the multigene paralogous families there seems to be one member under positive selection: RipH3, RipS7, RipG7. When we consider only 2 out of 3 LRTs for positive selection (see Additional file 7), we can define 14 more Rip coding sequences with evidence for positive selection, out of which 9 belong to the above-mentioned paralogous families (including RipA5). It is tempting to speculate that after duplications some of the paralogous genes could have undergone sub- or neo-functionalisation allowing the cognate Rip proteins to adapt to evolving plant targets or evade from host immunity.

### Comparative genomics and functional implications

#### The RSSC T3E core set: a large group of conserved effectors

The establishment of a near-complete T3E repertoire in strains representative of the large phylogenetic diversity of the RSCC allows a more specific and accurate comparison than those based on comparative genomic hybridizations [12]. We performed T3E repertoire comparisons using the following criteria: (i) *rip* genes listed as pseudogenes in the database were considered non-functional but those listed as containing frameshifts were considered as functional genes. The assumption that all the frameshifts are due to sequencing errors is probably an overestimation. Since we can’t validate this experimentally, and considering that the number of frameshifts identified is inversely correlated with the genomic sequence quality, we will keep this assumption. This is exemplified with GM1000 and CFBP2957 high quality genomes, not containing a single frameshift mutation in their T3E genes. (ii) The 16 hypothetical T3E newly identified in the different strains were also included in the repertoire for comparisons.

**Figure 3:**
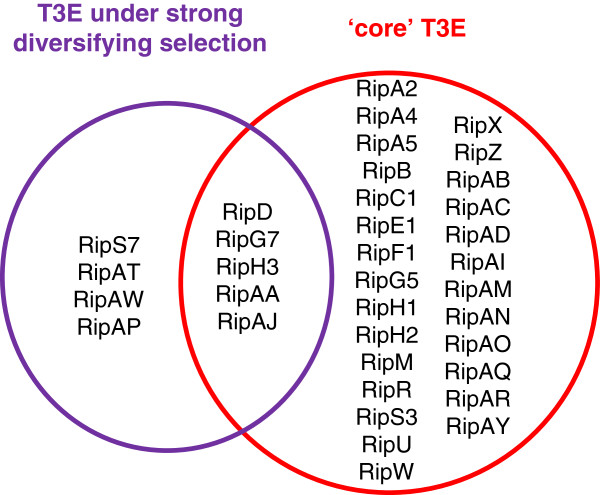
**Grouping of T3E *rip *** **genes. Circles group (i) genes under strong positive selection, (see Table**2**) and (ii) genes belonging to the core group of T3E conserved in 10 out of the 11 RSSC genome sequences.**

The RSSC is divided in three main phylogenetic clades corresponding to phylotypes 2, 4 and (1 + 3) [1,11]. A first comparison showed that 22 Rip gene families are present in the 11 strains studied. When the presence requirement is lowered at 10 out of the 11 strains, the number of gene families reaches 32 (Additional file 8). Considering that the event of loss of specific T3E genes in some strain lineages is possible (see for instance the significantly reduced repertoire of *R. syzygii* R24 or BDBR229), we believe that these 32 T3E are a good estimation of the subset of T3E probably present in the ancestral *R. solanacearum* strain. Interestingly, 5 out of 9 T3E genes families showing a strong signal of diversifying selection also belong to the core effector group (Figure 3). It is also interesting to notice that distinct members of paralogous Rip families (RipA, RipG and RipH) are also conserved among the 11 analyzed strains, indicating that duplications followed by differential evolution of these genes took place early before phylotype divergence [17]. The estimate of 32 core T3E certainly reflects the abundance of T3E in the *R. solanacearum* and, considering its genetic diversity as a species complex, appears significantly higher than the core list identified in *P. syringae* which is only 5 among 19 strains [13]. *R. solanacearum* ancestor presumably possessed more than 20 T3E, which were possibly acquired from the bacterial and phage communities in the soil or aquatic reservoirs.

#### T3E repertoire comparisons provide no clues on host specificity determinants

**Figure 4:**
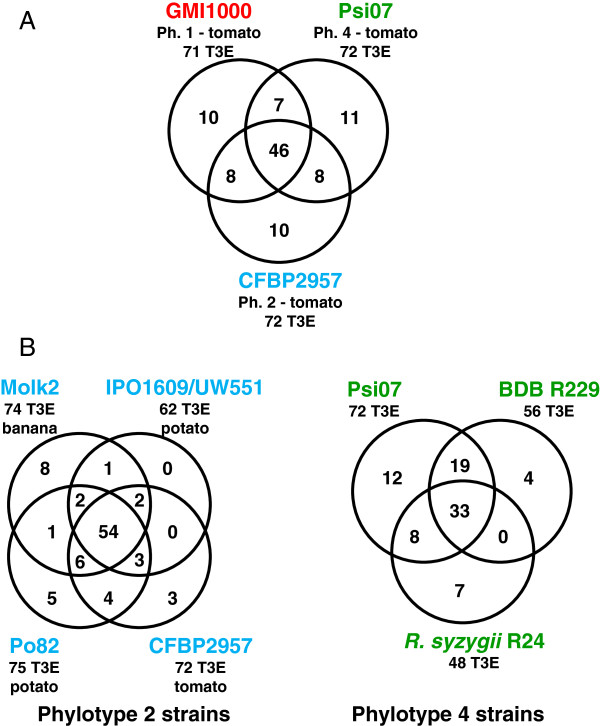
**T3E distribution in RSSC strains. RSSC strains are color-coded according to their phylotype goupe: Red for phylotype 1 and 3; Blue for phylotype 2 and green for phylotype 4 and related strains. A**. Shared T3E between representative strains of the three main phylogenetic lineages of the RSSC, all isolated from tomato. **B**. Shared T3E between *R. solanacearum* strains belonging to phylotype 2. The almost identical repertoires from strains IPO1609 and UW551 were merged for this comparison. **C**. Shared T3E between strains belonging to phylotype 4.

Phylotypes 1–3, 2 and 4 are the main genetic groups structuring the RSSC [1,11], A comparison of the T3E repertoires (also taking into account the 16 candidate genes) from GMI1000 (Phylotype 1), CFBP2957 (phylotype 2) and Psi07 (phylotype 4), representing the three species clades and all isolated from tomato, reveals a diversity of 100 T3E genes, almost half of which (47) are conserved among the three strains whereas one third (30 T3E) appears to be strain-specific (Figure 4A). This confirms that a majority of T3E are widely conserved in this species complex but also shows that the strain repertoires are also diversified, as observed in *P. syringae*[13] or *Xanthomonas* sp. [55].

*R. solanacearum* strains exhibit great variations in host range [4] and it is tempting to speculate that T3E repertoires shape these host range capabilities. In order to tentatively identify candidate genes involved in host specificity, we performed T3E repertoire comparisons within specific phylogenetic groups such as phylotype 2 or 4 using strains with marked host range differences (Figure 4B). These comparisons identified strain-specific genes but did not pinpoint strong host-specificity candidates. Indeed, none of the Molk2 specific T3E is common with those of the BDBR229 strain which is also pathogenic on banana; the same is true for potato-associated T3E genes from the Po82 and UW551/IPO1609 strains. Although more genomic sequences of RSSC strains are needed to perform robust associations between host range and T3E repertoires, these observations already suggest that host-range maybe controlled by multiple or differential combinations of T3E determinants, or determinants others than T3E, or that differences in T3E protein sequence or gene expression might also be involved [10]. Similar observations were reported for comparison of *P. syringae* pathovars T3E repertoires [56], thus reinforcing the idea that a complex genetic basis underlies host range evolution in plant pathogens.

Finally, intra-phylotype comparisons suggest that the proportion of conserved T3E is higher in phylotype 2 than in phylotype 4 strains (Figure 4C). Although phylotype 4 strains BDBR29 and R24 have undergone gene reduction potentially affecting this comparison, we still believe that this difference reflects the highest genetic diversity within phylotype 4 [9] and could also be associated with the diverse lifestyle among phylotype 4 strains [11].

#### Identification of novel T3E gene harboring putative ubiquitin-ligase domains

Molecular functions of most *R. solanacearum* T3E remain unknown, and more than half of the repertoire corresponds to proteins with no structural motif or domain suggestive of function. The search for functional motifs identified two T3E proteins, RipAR and RipAW, carrying a C-terminal domain structurally related to the *Shigella flexneri* IpaH ubiquitin ligase domain [57]. Although the overall similarity between IpaH and RipAR/RipAW is low, these *R. solanacearum* T3E have a C-terminal domain with a predicted structure consisting of 12 alpha-helices as determined for IpaH family proteins [57]. Most of the highly conserved residues in the IpaH family, including a highly conserved cysteine residue essential for activity [57], are conserved in RipAR and RipAW see sequence alignment in Additional file 9. Considering the previously identified T3E RipV, a *Salmonella* SspH1 homologue [58], and the RipG family members [16], *R. solanacearum* potentially harbors a total of 10 T3E endowed with potential ubiquitin-ligase activity. This highlights the probable central mechanism consisting in subversion of the host’s ubiquitination system by T3E during plant pathogenesis [59,60].

#### The specific case of the RipF translocon proteins

**Figure 5:**
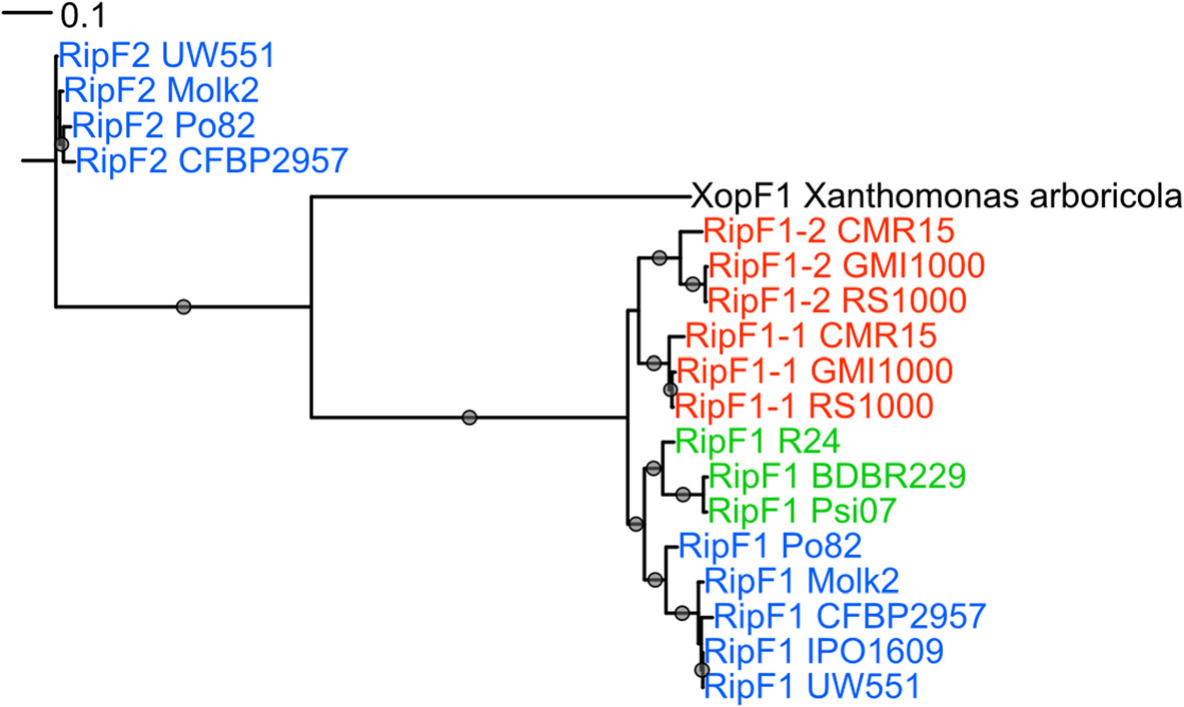
**Phylogenetic reconstruction of the RipF family.** PhyML phylogenetic reconstruction of the RipF family. The XopF from *Xanthomonas arboricola* [GenBank: AFV80105] is also included in this analysis. *R. solanacearum* GMI1000 RipF1_1 and RipF1_2 correspond to former PopF1 and PopF2.

The RSSC T3E list include RipF proteins (formerly PopF [35]) as substrates of the T3SS since they were identified as translocated into plant cells using the adenylate cyclase reporter assay [6]. RipF proteins are required for the translocation of other T3E and are T3SS translocator proteins presumably acting at the tip of the Hrp pilus and inserting into host cell membranes to permit T3E translocation [35,61]. Contrary to the structural components of the T3SS (including the Hrp pilus structural pilin) which are strongly conserved among all the strains from the RSSC analyzed to date, a comparative analysis of RipF revealed major differences among the currently sequenced RSSC strains. Strains belonging to phylotypes 1, 2 and 3 possess two RipF whereas strains from phylotype 4 have only one (RipF1) as *Xanthomonas* spp. In phylotypes 1 and 3 the second gene, formerly named PopF2 [35], is phylogenetically close to the first one named PopF1. However in phylotype 2, the second gene product belongs to a distinct phylogenetic branch, suggesting an ancient divergence from the other RipF1/PopF1 lineage. These observations incited us to rename GMI1000 PopF2 as RipF1_2 (PopF1 being RipF1_1) whereas RipF2 is proposed to designate the gene from the phylotype 2 (see Figure 5). This peculiar evolutionary history of the RipF family makes this one of the most stringent discriminating probe among all Rip genes for distinguishing the three main phylotype groups of the RSSC.

The biological implications of this gene duplication of the RipF translocator in some RSSC lineages and the structural divergence between the RipF1/RipF2 family members are unknown. In GMI1000, RipF1_1 has a major role in T3E translocation in tomato and tobacco whereas RipF1_2 plays a minor role in this process on these hosts [35]. The specific involvement of RipF2 and RipF1 in pathogenicity of phylotype 2 strains will need to be addressed in future studies.

## Conclusion

T3E are essential to *R. solanacearum* pathogenesis but progress in understanding of their relative contribution to disease through reverse genetic approaches has been hampered by the evidence of functional redundancies, due to the existence of large T3E repertoires. In this study, we have undertaken groundwork for a global inventory of *R. solanacearum* T3E at the species level in order to provide to the community a curated dataset, tools and a rationalized nomenclature that should pave the way for future work on RSSC effectomics. We conducted a large scale approach aimed at the identification, expert annotation and phylogenetic analyses of T3E from the RSSC, a species complex showing considerable genomic diversity [10,11] and responsible for one of the most devastating bacterial disease of plants worldwide [2]. Our search yielded a total of 94 T3E Rip genes and 16 additional candidate T3E genes distributed among the 11 genomes analyzed in this study. This total of more than 100 predicted T3Es is significantly higher than the T3E inventories from other bacterial plant pathogens. Indeed, in *P. syringae*, genome analysis of 19 phylogenetically diverse isolates revealed the existence of 58 T3E genes [13] (the online resource http://www.pseudomonas-syringae.org, references 61 Hop orthologous groups) whereas this number is estimated to 52 in *Xanthomonas* spp [55]. These comparisons highlight the great diversity of T3E genes present in the RSSC and the apparent complexity of T3SS-dependent pathogenesis in this species complex.

The RSSC T3E also appears to be highly dynamic, as evidenced by the number of T3E under positive selection indicative of possible neo-functionalization or the number of T3E pseudogenes identified in this study. In particular, there is an obvious tendency to T3E gene decay in *R. syzygii* which is correlated with the genome reduction in this strain [11]. *R. syzygii* is an exception among the RSSC since it is strictly limited to Clover tree, the T3E repertoire reduction in this strain may be a consequence of this host specialization. On the other hand, the cornucopia of T3E identified in *R. solanacearum* and other related pathogenic beta-proteobacteria is probably a factor explaining the exceptional adaptation of these pathogens to such a wide diversity of hosts. Importantly, phylogenetic analyses allowed the definition of novel T3E genes, resulting in the definition of new Rip genes orthologous group or paralogs of already identified Rip genes. It is conceivable that these newly defined groups correspond to T3E genes with novel functional specificities.

Our analysis should also be helpful for refined functional studies: (i) the RipF1-RipF2 translocon proteins appear as major discriminating determinants among the main lineages of the RSSC and this probably reflects a fundamental evolutionary divergence (ii) global comparisons of repertoires among genetically diverse strains identified a set of 20–30 core T3E widely distributed in the species which could presumably be considered as ancestral T3E important in the interaction of the pathogen with its hosts, and (iii) the identification of T3E displaying a positive selection pattern may provide hints on the determinants evolving under plant selection pressure, (iv) our bioinformatics pipeline is dedicated to rapidly predict and assign Rip identifiers to all homologous T3E genes in newly sequenced strains of the RSSC.

## Methods

### Data sources

General information of the features of the 14 strains of the RSSC and the corresponding genome sequences used for T3E mining is provided in Additional file 10. These strains are representatives of the RSSC in terms of host range, worldwide geographic origin and phylogenetic distribution [10,11].

### T3E inventory and annotation in RSSC genomes

PatScan searches [62] for the hrp_II_ box element (TTCGn_16_TTCG) were performed in RSSC genomes using the criteria previously used [24], *i.e.*: one mismatch allowed, considering only hits in the 500 bp region upstream of a start codon. Analysis of the 50 amino acid N-terminal domain of candidate T3E for detection of T3SS-dependent export pattern was made using the criteria defined previously [5], which considered as positive a N-terminal domain meeting at least two out of the three following rules: (i) content in Serine + Proline >30%, (ii) content in Leucine <10% and (iii) absence of acidic residues within the first twelve amino acids.

### Prediction of T3E start codon

We observed a great heterogeneity among the predicted start codons for many T3E families in the RSSC annotated genomes deposited at GenBank. When possible, multiple sequence alignments of the regions located downstream the hrp_II_ box element were performed to predict the most probable start codon which was defined as the more distal 5′ initiator codon conserved among the different strain sequences.

### Frameshift and pseudogene prediction

T3E genes were annotated as frameshift in two cases: (i) when several contiguous open reading frames displayed homology to a defined Rip gene sequence (thus resulting in the annotation of two or multiple gene fragments), and (ii) when the T3E gene sequence was located on a contig border (thus resulting in the annotation of a T3E gene fragment).

T3E genes were defined as pseudogenes in the following situations: (i) the structure of T3E gene was strongly altered with a gene size <50% to other known alleles, or led to the deletion of the N-terminal domain necessary for T3SS-dependent translocation, (ii) the T3E gene open reading frames was disrupted by the insertion of an IS element, or (iii) there was experimental evidence that the T3E gene product is not translocated or secreted by the T3SS.

### Detection of candidate effectors in sequenced genomes using “ScanYourGenome”

The first step of the pipeline we developed to detect putative effector candidates is a *de novo* proteome prediction. To achieve this, we run a blastx of the genome against the T3E proteins and use this data as an input of the prokaryotic gene predictor FrameD [63]. This tool is run twice with the T3E nucleic coding sequence as model: the first pass is done with a high frameshift penalty score and the second one with a lower one, allowing frameshift and pseudogene prediction. To ensure the completeness of this new effectome, we add translated regions matching a T3E member according to the blastx results.

The second step of the pipeline is the search of homologous T3E member for each candidate. In order to get the best precision, we run different methods and synthesise information taking into account the specificity of each method and parameters.

The first method is the search for homology using a modified version of OrthoMCL [64] pipeline. The modifications used are: filter inactivation in the blastp preprocess with default parameters and stepwise decrease of the percent match cutoff (from 90% to 60%) in ortholog clustering in order to retrieve shorter pseudogene. The best blastp, hmmscan and tblastn are respectively kept in order to complete orthoMCL assignation or to remove ambiguity of multiple assignations, especially in the case of paralogous gene families.

The results are ordered according to the stringency of the method (from OrthoMCL90 > OrthoMCL80 > OrthoMCL70 > OrthoMCL60 > blastp > HMMscan > tblastn). It is also indicated whether a frameshift mutation was introduced to produce a better homologous sequence. If the candidate gene is shorter than 80% of the average length of the cognate Rip gene, then the gene is tagged as a candidate.

This pipeline, written in Perl, is available through the T3E web interface and all parameters are available on demand.

### Phylogeny

Rip sequences were aligned using the ProGraphMSA program, which implements the evolution-aware alignment [65,66]. This program performs well with indel rich data as well as with variation in tandem repeats such as leucine rich repeats, as is often the case here. All phylogenies were reconstructed using fast maximum likelihood (ML) heuristic search. For all individual Rip genes we captured information from both nonsynonymous and synonymous sites by using tree searches under codon model M0 [67] using CodonPhyML [51].

Since phylogenies for paralogous gene families described much more diverse datasets, they were reconstructed under amino acid model LG [68] with C-rate variation among sites [69], as implemented in PhyMLv3.0 [70]. Branch supports were estimated using the aBayes method, which is fast, accurate and has performance comparable with the Bayesian method [71]. Phylogenetic trees were produced using the online software ITOL [72].

### Analysis of selection pressures

Selection pressures were analysed on T3E genes datasets containing three or more orthologs. Selection pressures on T3E genes were evaluated using Markov models of codon substitution, and three pairs of likelihood ratio tests (LRTs) were used to detect positive selection like previously described [17].

### Testing for recombination

The same data used for the selection pressure analysis were used to estimate the population recombination rates using the approximate-likelihood coalescent method and permutation test [73] like previously described [17].

### Availability of supporting data

All the data present in this work and supporting our analysis is available on the publicly accessible database that has been set up and will be maintained by us.

https://iant.toulouse.inra.fr/T3E is a website designed to provide the user with a convenient and straightforward access to all the underlying data.

### GenBank accessions

Out of the 841 *Ralstonia solanacearum* accessions used in this study, we have submitted 42 new and proposed the modification of the annotation of 289 other individual T3E gene accessions to GenBank. All the Genbank accessions appear on the database webpage (under data/supplementary data and also as Additional file 11.

## Abbreviations

BDB: Blood disease bacterium; HGT: Horizontal gene transfer; IS: Insertion sequence; LRT: Likelihood ratio test; ML: Maximum likelihood; RIP: *Ralstonia* injected protein; RSSC: *Ralstonia solanacearum* species complex; T3E: Type III effector; T3SS: Type III secretion system.

## Competing interests

The authors declare no competing interests.

## Authors’ contributions

NP, SC and SG designed the study. SC performed and structured all the bioinformatics pipeline and database; MA performed the selection and recombination analysis as well as the phylogenetic reconstructions. NP, LP, ACC and SG participated in the curation of the data. NP, MA, SG analysed the data. NP and SG wrote de paper. All authors have read and approved the manuscript for publication.

## Supplementary Material

Additional file 1 Table displaying the additional 16 T3E candidates in the RSSC. Click here for file

Additional file 2 **List of T3E genes identified in the 11 strains of the RSSC used in this study.** The result of “ScanYourGenome” on three additional strains (K60, FQY_4 and Y45) is also presented. Click here for file

Additional file 3 Experimental validation of type III dependent secretion of RipAM. Click here for file

Additional file 4 Phylogenetic reconstruction for all paralogous T3E genes together with selected homologs from other bacteria. Click here for file

Additional file 5 **Phylogenetic tree reconstruction of T3E with proven (YopJ, RipP2**_**GMI1000**_**) and possible acetyl-transferase activity.** Click here for file

Additional file 6 List of T3E orthologues with GC% bias and association with mobile elements. Click here for file

Additional file 7 Table displaying the calculated positive selection and recombination probabilities for the whole T3E dataset. Click here for file

Additional file 8 List of the 32 core T3E presented in this study. Click here for file

Additional file 9 Sequence alignment of RipAR and RipAW C-terminal domains with IpaH ubiquitin ligases. Click here for file

Additional file 10 List and features of RSSC strains and corresponding genomic sequences used in this study. Click here for file

Additional file 11 List of all accessions used in this work. Click here for file
