# Supplementary material for: Repertoire, unified nomenclature and evolution of the Type III effector gene set in the Ralstonia solanacearum species complex
Source: BMC Genomics. 2013 Dec 6;14:859. doi: 10.1186/1471-2164-14-859 (PMC3878972; doi:10.1186/1471-2164-14-859)
Supplement: Additional file 1 — Table displaying the additional 16 T3E candidates in the RSSC. [file 1471-2164-14-859-S1.doc]

| **Hypothetical T3E family** | **Representative gene member**^§^ | ***hrp_II_* box in promoter*** | **Number of strains harbouring one family member^#^** | **Features / Functional domain or motif** |
| --- | --- | --- | --- | --- |
| RS_T3E_Hyp1 | Psi07 RSPsi07_0331 | yes | 5 |  |
| RS_T3E_Hyp2 | Psi07 RSPsi07_1883 | yes | 2 |  |
| RS_T3E_Hyp3 | Psi07 RSPsi07_mp0834 | yes | 3 |  |
| RS_T3E_Hyp4 | Psi07 RSPsi07_mp1047 | yes | 4 [1] |  |
| RS_T3E_Hyp5 | Psi07 RSPsi07_mp1559 | yes | 2 |  |
| RS_T3E_Hyp6 | CMR15 CMR15v4_30001 | yes | 1 |  |
| RS_T3E_Hyp7 | Molk2 RSMK06225 | yes | 2 |  |
| RS_T3E_Hyp8 | Molk2 RSMK02655 | yes | 4 | Repeat domain |
| RS_T3E_Hyp9 | UW551 RRSL_01783 | yes | 4 [1] |  |
| RS_T3E_Hyp10 | Molk2 RSMK02638 | yes | 1 [1] | Repeat domain |
| RS_T3E_Hyp11 | Molk2 RSMK01187 | yes | 2 [2] |  |
| RS_T3E_Hyp12 | Molk2 RSMK03335 | yes | 4 [1] |  |
| RS_T3E_Hyp13 | Po82 RSPO_m01098 | yes | 1 |  |
| RS_T3E_Hyp14 | R229 BDB mp_40006 | yes | 1 |  |
| RS_T3E_Hyp15 | Psi07 RSPsi07_1860 | yes | 3 [1] | Serine/Threonine kinase domain |
| RS_T3E_Hyp16 | GMI1000 RSc3174 | yes | 3 |  |

^§^ Gene designation follows the strain name

* Canonical *hrp_II_* box detected in at least one promoter of a gene family member

**^#^** Number in brackets corresponds to probable pseudogene(s)
