# Supplementary material for: Repertoire, unified nomenclature and evolution of the Type III effector gene set in the Ralstonia solanacearum species complex
Source: BMC Genomics. 2013 Dec 6;14:859. doi: 10.1186/1471-2164-14-859 (PMC3878972; doi:10.1186/1471-2164-14-859)
Supplement: Additional file 3 — Experimental validation of type III dependent secretion of RipAM. [file 1471-2164-14-859-S3.docx]

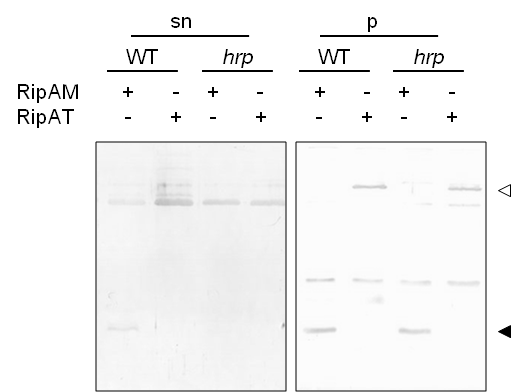


**Additional file 10. Experimental validation of type III dependent secretion of RipAM.**

GMI1000 and GMI1694 (hrcV mutant [1]) strains were transformed with plasmids carrying RSc3272 RipAM (pACC620) and Rsp1388 RipAT (pACC621), these plasmids were generated by LR-recombining pENTRY vectors (carrying both ORF) into a dedicated destination vector pNP329 derived from pRC [2], allowing the expression of RipAM-3HA and RipAT-3HA under the RipG7 promoter. After secretion assay [1], the supernatant (sn) and bacterial pellet (p) proteins were analyzed by western blot against the HA tag using NBT/BCIP staining. RipAT is annotated as being pseudogene in GMI1000 because of an N-terminal truncation, this status is confirmed here by the absence of secretion of the corresponding protein.

1. Cunnac S, Occhialini A, Barberis P, Boucher C, Genin S: Inventory and functional analysis of the large Hrp regulon in Ralstonia solanacearum: identification of novel effector proteins translocated to plant host cells through the type III secretion system. *Mol Microbiol* 2004, 53:115–128.

2. Monteiro F, Solé M, van Dijk I, Valls M: A chromosomal insertion toolbox for promoter probing, mutant complementation, and pathogenicity studies in Ralstonia solanacearum. *Mol Plant-Microbe Interactions MPMI* 2012, 25:557–568.
