# Supplementary figures and images for: Repertoire, unified nomenclature and evolution of the Type III effector gene set in the Ralstonia solanacearum species complex
Source: BMC Genomics. 2013 Dec 6;14:859. doi: 10.1186/1471-2164-14-859 (PMC3878972; doi:10.1186/1471-2164-14-859)

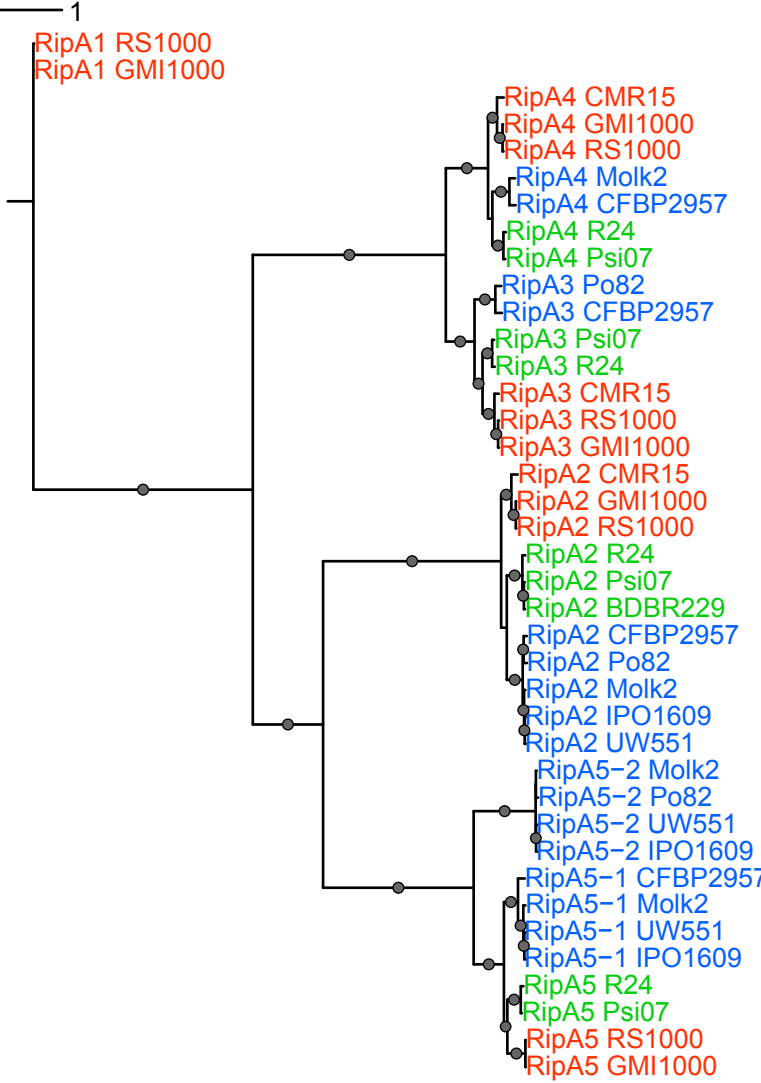

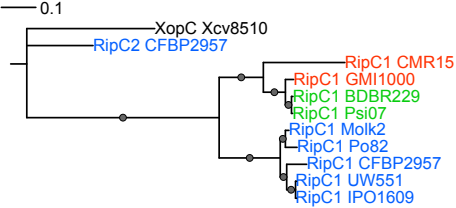

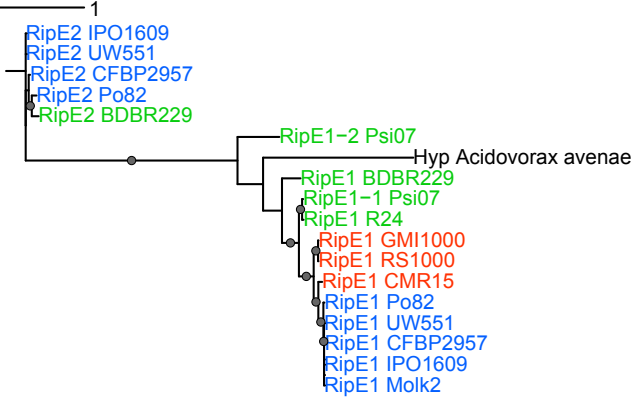

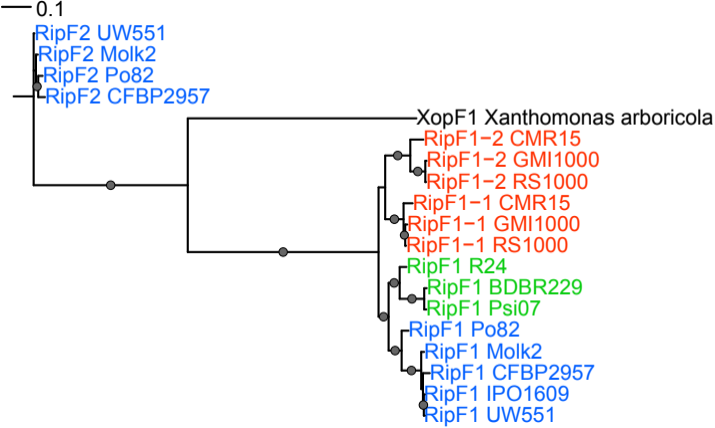

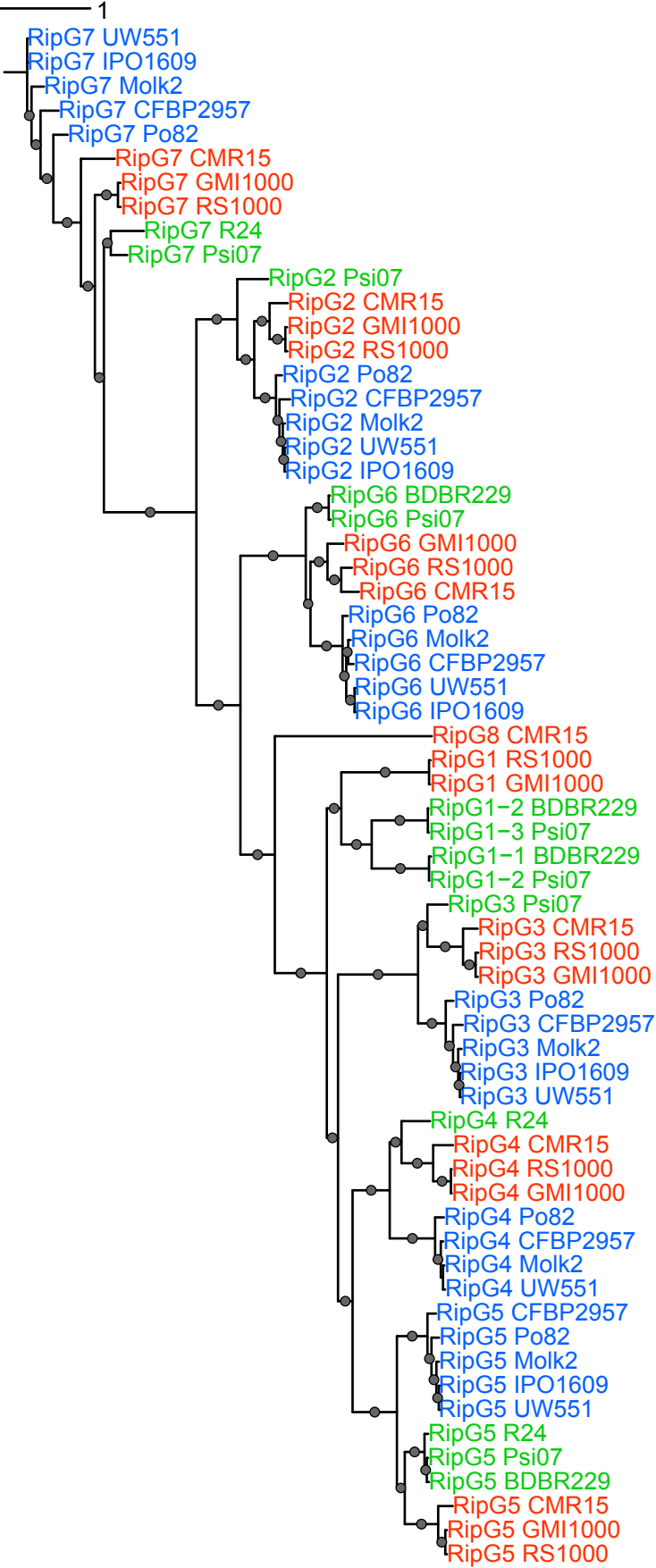

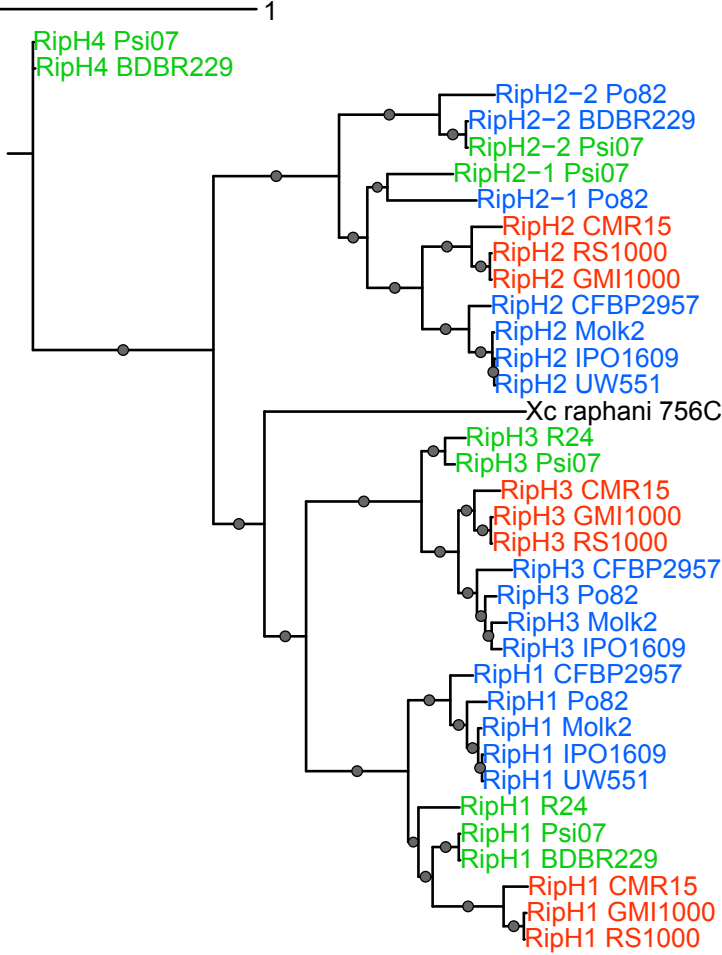

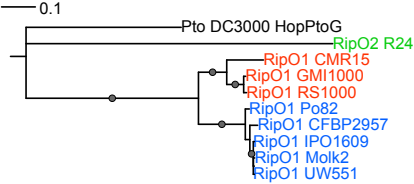

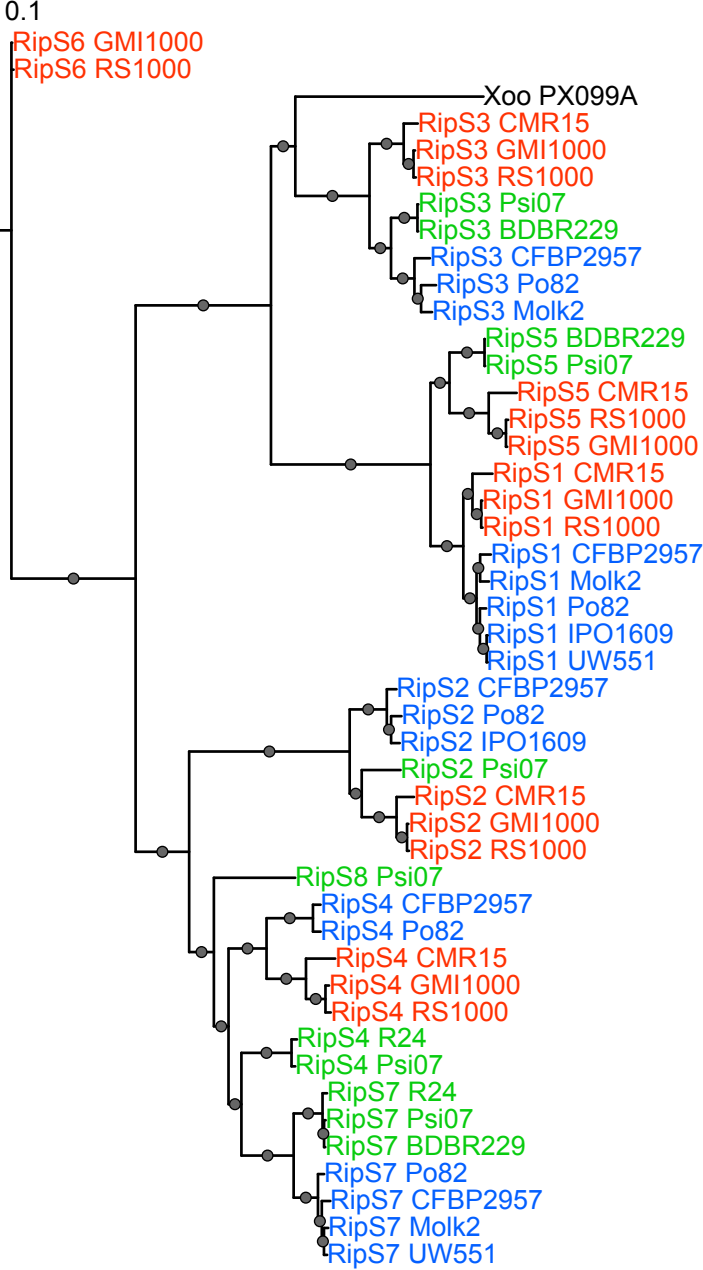

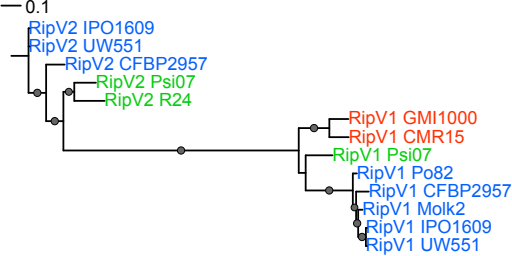

— 0.1

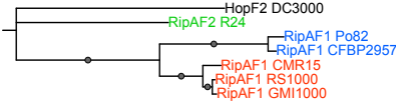

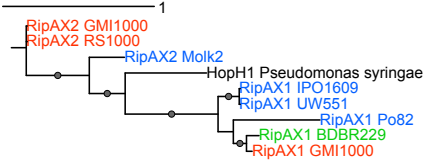

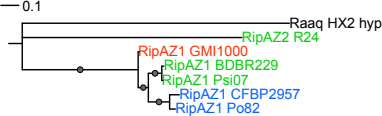

Supplement: Additional file 4 — Phylogenetic reconstruction for all paralogous T3E genes together with selected homologs from other bacteria. [file 1471-2164-14-859-S4.pdf]

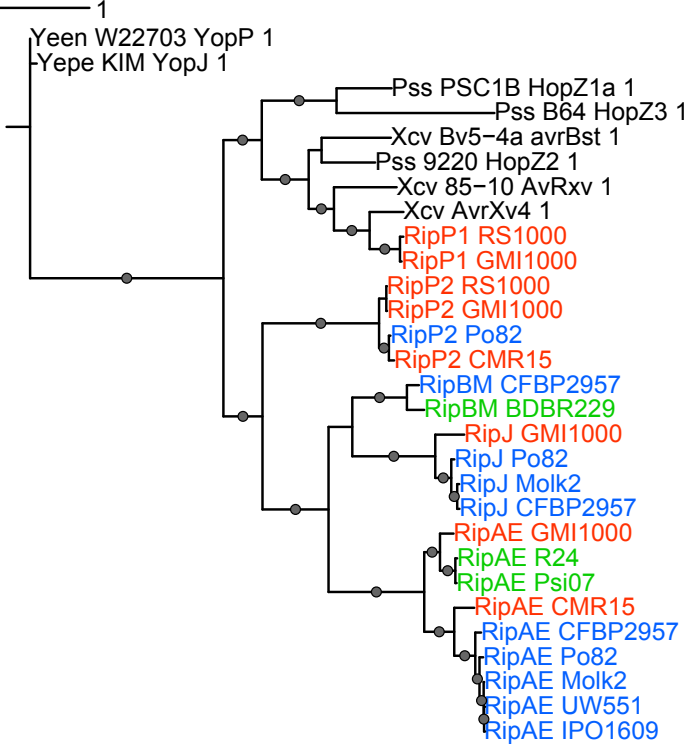

Supplement: Additional file 5 — Phylogenetic tree reconstruction of T3E with proven (YopJ, RipP2 GMI1000 ) and possible acetyl-transferase activity. [file 1471-2164-14-859-S5.pdf]
