# Supplementary material for: Repertoire, unified nomenclature and evolution of the Type III effector gene set in the Ralstonia solanacearum species complex
Source: BMC Genomics. 2013 Dec 6;14:859. doi: 10.1186/1471-2164-14-859 (PMC3878972; doi:10.1186/1471-2164-14-859)
Supplement: Additional file 6 — List of T3E orthologues with GC% bias and association with mobile elements. [file 1471-2164-14-859-S6.docx]

List of T3E genes displaying significant variation in percentage of guanine+cytosine content (GC%) compared to the mean content of the *R. solanacearum* species (67%). Physical association of these genes with mobile genetic elements such as bacteriophage sequences, Insertion Sequences (IS) or transposable elements is indicated.

Paralogous genes with probable inter-species HGT (*i.e* when phylogenetical analysis indicates that this gene is closer to a paralogue outside of the *R. solanacearum* species) are highlighted in red.

|  | **Mean GC% content** | **Physical association with mobile elements** |
| --- | --- | --- |
| RipO2 | 48 | + (transposase) |
| RipAF2 | 51 | + (phage integrase) |
| RipAX1 | 51-59 | - [GMI1000, Molk2]  + [Po82, BDB R224] (IS) |
| RipBE | 55 | unknown |
| RipP1 (PopP1) | 55-56 | + (bacteriophage) |
| RipBA | 55-56 | - |
| RipAX2 | 53-58 | + (bacteriophage) |
| RipE2 | 54-59 | - [IPO1609, CMR15]  + [Psi07, BDB R224] (IS), [Molk2, Po82] (bacteriophage) |
| RipT | 57-58 | + (bacteriophage) |
| RipBD | 58-59 | + (bacteriophage) |
| RipJ | 58-60 | - [GMI1000, CFBP2957, UW8551]  + [Molk2, Po82] (IS) |
| RipAZ2 | 59 | - |
| RipP2 (PopP2) | 59-60 | + (bacteriophage) |
| RipAZ1 | 59-61 | - |
| RipBG | 60 | - |
| RipO1 | 60-61 | - |
| RipAG | 61-62 | + (bacteriophage) |
| RipG8 | 62 | - |
| RipP3 (PopP3) | 62 | + (bacteriophage) |
| RipC2 | 61-63 | + (IS) |
| RipAA (AvrA) | 61-64 | - [GMI1000, Psi07, CMR15, CFBP2957, *R. syzygii* R24]  + [IPO1609, UW551, Molk2, Po82] (IS) |
| RipA3 | 70-72 | - |
| RipA4 | 70-72 | - |
| RipAM | 70-73 | - |
| RipM | 71-73 | - |
