# Supplementary material for: Repertoire, unified nomenclature and evolution of the Type III effector gene set in the Ralstonia solanacearum species complex
Source: BMC Genomics. 2013 Dec 6;14:859. doi: 10.1186/1471-2164-14-859 (PMC3878972; doi:10.1186/1471-2164-14-859)
Supplement: Additional file 8 — List of the 32 core T3E presented in this study. [file 1471-2164-14-859-S8.docx]

**The list of 22 core T3E conserved in all 11 strains from the RSSC**

| **T3E Rip family** | **Features** | **Homologues in other bacterial species** |
| --- | --- | --- |
| RipA2 | AWR family | *Acidovorax* and *Burkholderia* spp. |
| RipA4 | AWR family | *Acidovorax* and *Burkholderia* spp. |
| RipA5 | AWR family | *Acidovorax* and *Burkholderia* spp. |
| RipB | Nucleoside ribohydrolase domain | *P. syringae* HopQ1 |
| RipD |  | *P. syringae* HopD1 |
| RipE1 |  | *P. syringae* HopX |
| RipF1 | Translocon protein | *Xanthomonas* sp. HrpF |
| RipG5 | GALA family, F-box LRR domains |  |
| RipG7 | GALA family, F-box LRR domains |  |
| RipH2 | HLK family | *Xanthomonas* sp. XopP |
| RipR |  | *P. syringae* HopR |
| RipU |  |  |
| RipW | Harpin with pectate lyase domain | *P. syringae* HrpW |
| RipZ |  |  |
| RipAB | NLS-harboring protein |  |
| RipAC | LRR domain |  |
| RipAD |  |  |
| RipAI |  |  |
| RipAJ |  |  |
| RipAN |  |  |
| RipAO |  |  |
| RipAY |  |  |

**Additional 10 core T3E (conserved in 12/13 strains from the RSSC)**

| RipC1 | HAD phosphatase domain | *Xanthomonas* sp. XopC |
| --- | --- | --- |
| RipH1 | HLK family | *Xanthomonas* sp. XopP |
| RipH3 | HLK family | *Xanthomonas* sp. XopP |
| RipM |  |  |
| RipS3 | SKWP family | *P. syringae*  *Xanthomonas* sp. XopAD |
| RipX | Harpin | *P. syringae*  *Xanthomonas* sp. |
| RipAA | Avirulence protein recognized by *Nicotiana* sp. |  |
| RipAM |  |  |
| RipAQ |  |  |
| RipAR | Ubiquitin-ligase domain |  |
