# Supplementary material for: Repertoire, unified nomenclature and evolution of the Type III effector gene set in the Ralstonia solanacearum species complex
Source: BMC Genomics. 2013 Dec 6;14:859. doi: 10.1186/1471-2164-14-859 (PMC3878972; doi:10.1186/1471-2164-14-859)
Supplement: Additional file 9 — Sequence alignment of RipAR and RipAW C-terminal domains with IpaH ubiquitin ligases. [file 1471-2164-14-859-S9.docx]

*

1. SspH1 451-TENFKKDAGFKAQISSWLTQLAE--DAALRAKTFAMATEATSTCEDRVTHA

2. IpaH9.8 296-TVSARNTSGFREQVAAWLEKLSA--SAELRQQSFAVAADATESCEDRVALT

3. RipAW_CFBP2957 124--RPLHRAGSVRELGH--LLKLAARSDA-YRAFCFDIARGADADCHDNVDVI

4. RipAW_GMI1000 138--KEPHRADSVQELGA--VLKLAAHNDA-YRAFCFDVAGGADADCYDNAEVI

5. RipAW_Psi07 70--KTSHRAGSVRELGN--ILQLAACNDA-YRALCFDLAGGADANCHDNVDVI

6. RipAR_GMI1000 283--RPAHRAESLKELGG--ILQLAAHNDA-YRTRCFDICGGADANCHDNVDVI

7. RipAR_Molk2 381-LQPEHRAASVQELGT--ILTLVAHNDA-YRASCFDICGGAEADCHDNVDVV

8. RipAR_Psi07 305--WPQHRAGSMQELGS--ILELAAHNDA-YRAFCFNLCGGADANCHDNVDVI

.. . . . .

1. LHQMNNVQLVHNAEKGEYDNNLQGLVSTGREMFRLATLEQIAREKAGTLALVDDVEVYLAFQNKLKESLELT

2. WNNLRKTLLVHQASEGLFDNDTGALLSLGREMFRLEILEDIARDKVRTLHFVDEIEVYLAFQTMLAEKLQLS

3. FGNLRLAARNPTYHGNA----SLDQVLKYHKSCVPWSLIDD-FVSKQFSLFDEPLEKVLALRIRLSDILPVK

4. FGNLRLAARDPTYHGNA----SLEQVLNYHKRCVPWSLVDD-FVSERFPLFAESLENVLALRIRLSDILPIR

5. FGNLRLAAKDPTYHGNA----GLDQVLKYHKSCVPWSLIDD-FVSTRFPSFDESLERVLALRIRLSDLLPIK

6. FGNLRLAARDPSYHGNA----QLHEVLSYHNRCMPWTLIDD-FVSHRFGRGDQ-LERALALRVRLSDILPIT

7. FGNLRLAARDPALRGNA----PLSEILSYHKFCVPWTLIND-FVSHRFSSGDQ-LEKVLALRIRLSDILLVK

8. FGNLRLAARDPSYRGNA----GLHEILDYHNRCVPWTLIDD-FVSHRFAQGDL-LEKVLALRIRLPDILPIT

. .. .. . . .. . . .

1. SVTSEMRFFDVSGVTVSDLQAAELQVKTAENSGFSKWILQW-GP-LHSVLERKVPERFNALREKQISDYEDT

2. TAVKEMRFYGVSGVTANDLRTAEAMVRSREENEFTDWFSLW-GP-WHAVLKRTEADRWAQAEEQKYEMLENE

3. T--PAMVNRVLANVDDEAEKQARAYIATHRRTKEHLQRNLCRSPAWHRFLVQRHPVEFAANTLLWDAALQDV

4. T--PAMTFDNMTSVNQGVEAQARAYIARHCDREAKLQRNLCRSPAWRQFMERQHPVEFTANTLLWASALQAV

5. T--PAMCFSGIAGVDRAIEKQARAYIAAHRKTEACLQRSLTRSPAWRQFLAQRYPVEFVANTLLWESALQDV

6. T--PAMLHHDIARIDNAHEREARAYISAHLGTQEHLLRNLSRSPAWRQFLEQQRPVEFAANTLLWESALQDV

7. T--PAMLYSRLADITDAHELEARHYINTHVGTEANLLRSLSRSPTWRTFLTQRHPVEFAANTLLWSAALEEL

8. T--PAMLHGPVAGIDDAHELEARSYINAHLGTPENLLRSLSRSPAWRPFLEQRHPVEFVANTLLWDAALQDL

. . . . . .

1. YRKLYDEVLKSSGLVDD---------------TDAERTIGVSAMDSAKKEFLDGLRALVDEVLGSY -692

2. YPQRVADRLKASGLSGD---------------ADAEREAGAQVMRETEQQIYRQLTDEVLALRLSE -537

3. MKKADDGATSRSAQPHPDTDA-LGSRTEALARARAMPGIGTGRAFQRLQENATVCLMEGMTRRLVV -367

4. M-EQRPEGAAMAVPPEVNTVS-FGSRTEALARARAMPGIGTGHAFRHLQQNATVLLSEDLTRRLVV -385

5. SGKTQDDGSARGTPPSVDTES-HGSRTEMLARARAMPGIGTGRAFQRLQGNATLLLMEDMTRRLVA -318

6. MAKPAGDG-AVADAPRAASTTAPGSRTEALAQAHAMPGIGTGQAFQHRQENATAMLAETMTRKLVM -530

7. GAKSAS-GSAGPAQPAGNPAA-FGSRTEALAQARAMPSLGTGLAFRHLQENATVMVMEAMTHKLVA -628

8. MAKPAGGGSAAATQPARDAAS-FGSRTEALAQARAMPGIGTGKAFQHLQENATAMMVETMTRRLVA -552

Additional file 8: **Multiple sequence alignment of the C-terminal domains of RipAR and RipAW homologs.** A set of six representative proteins was selected from the alignment of 17 RipAR and RipAW family proteins and compared to the ubiquitin ligase domain of SspH1 from *Salmonella typhimurium* (1) and IpaH9.8 from *Shigella flexneri* (2). Alignment includes sequences from RipAW of strains CFBP2957 [phylotype 2] (3), GMI1000 [phylotype 1] (4) and Psi07 [phylotype 4] (5) and from RipAR of strains GMI1000 (6), Molk2 [phylotype 2] (7) and Psi07 (8). Residues that are identical in all or most sequences are highlighted in red and conserved identical residues within *R. solanacearum* sequences are highlighted in blue. Residues conserved in all or most members of the IpaH/SspH1 family (Singer *et al*. 2008) are indicated at the bottom by a purple dot. The critical cysteine residue in position 337 of IpaH9.8 which is essential for ubiquitin ligase activity (Singer *et al*. 2008) is indicated by an asterisk.
